# The role of ginseng in aging: Insights into regulatory T cells activation and mitochondrial regulation

**DOI:** 10.1016/j.jgr.2025.05.005

**Published:** 2025-05-16

**Authors:** Hamid Iqbal, Dong-Kwon Rhee

**Affiliations:** a Department of Pharmacy, CECOS University, Peshawar, Pakistan; b School of Pharmacy, Sungkyunkwan University, Suwon, Republic of Korea

**Keywords:** Aging, Ginseng, Inflammaging, Mitochondria, Regulatory T cells

## Abstract

A hallmark of aging is the progressive decline in resilience to stress and mitochondrial activity. As mitochondrial function decreases with aging, mitochondrial DNA (mtDNA) is shed under apoptotic stress, resulting in a persistent low-level of sterile inflammation (called inflammaging) that induces the aging program. In response to inflammaging, the body activates a compensatory anti-inflammatory response, including the activation of regulatory T (Treg) cells, to prevent excessive tissue damage. Recent studies have highlighted the dysfunction of Treg cells in elderly patients, suggesting that their critical role in the mitigation of aging. Additionally, mitochondrial electron transport chain (ETC) complexes, particularly complexes II and III, are essential for the function of Th1 and Treg cells, respectively. Since centenarians experience less inflammaging, this review aims to explore the anti-aging properties of ginseng. Research has shown that ginseng and its active compounds, ginsenosides, increase Treg cells population in aged mice and convert pro-inflammatory M1 macrophages into anti-inflammatory M2 macrophages. Furthermore, ginseng enhances antioxidant protein expression, decreases reactive oxygen species (ROS) production, restores mitochondrial ATP and membrane potential, and exerts anti-aging effects. Ginseng has been shown to extend lifespan, promote beneficial gut bacteria, and slow cognitive decline through its influence on immune cell circulation. Future research, including clinical trials, is needed to clarify the regulatory effects of ginseng on Treg cells, mitochondrial complexes, and their associated metabolites, as well as the interconnected mechanisms between them.

## Graphical abstract

Schematic representation of the therapeutic intervention of ginseng to attenuate age-related diseases using *in vivo* and *in vitro* models.

**Figure:**
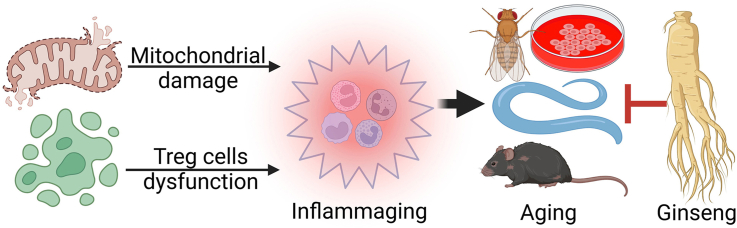

## 1 Introduction

Aging is a gradual and irreversible but delayable biological process affecting all organisms that involves complex structural and physiological damage and functional decline to the body as well [1,2]. With the growing global population, the deterioration of health associated with aging has garnered significant attention due to its profound effects on the healthcare system [2,3]. Together, age-related pathologies have negative impacts on health and social life of individuals, families thus raising the concerns of morbidity and mortality [1,3].

The remodeling of the immune system which includes chronic low-grade inflammation is a major hallmark of the aging process and many age-related pathologies [[2], [3], [4], [5]]. This immune state, often referred to as inflammaging [4,6], is marked by elevated levels of pro-inflammatory mediators in the blood, such as C-reactive protein (CRP), tumor necrosis factor (TNF)-α, and interleukin (IL)-6 [2,4,5,7]. These pro-inflammatory markers are closely linked to longevity, aging-related diseases, and are positively associated with mortality [1,2,4]. In contrast, older adults generally show reduced levels of anti-inflammatory cytokines in their plasma. In centenarians, however, the levels of CRP, IL-12, TNF-α, interferon-gamma (IFN-γ), IL-6, and IL-10 are higher compared to those in older adults, while the levels of IL-17A, IL-1β, IL-23, and transforming growth factor-β (TGF-β) in centenarians resemble those seen in younger adults [4].

The Nuclear factor-κB (NF-κB), a key regulator of inflammation and a responsive transcription factor to redox signals, plays a crucial role in both inflammation and cellular senescence, with its activation being triggered by oxidative stress [[8], [9], [10]]. The accumulation of senescent cells in tissues and organs is a key focus in aging research, as these cells play a pivotal role in the mechanisms of aging [6,7,11,12]. Several age-related stresses induce the accumulation of senescent cells into tissues. The pro-inflammatory phenotype of senescent cells enhances the recruitment of immune cells into aging tissues. The inflammaging state evokes compensatory immunosuppression which counteracts the low-grade inflammation present in aged tissues. Consequently, immunosuppression impairs the clearance of senescent cells, i.e., it enhances inflammaging and immunosuppression. Increased immunosuppression with aging impairs the maintenance of tissue homeostasis and induces the degenerative changes evident in aging tissues. Interestingly, senescent cells display a secretory phenotype and thus this state has been called the senescence associated secretory phenotype (SASP). It seems that senescent cells secrete pro-inflammatory factors to alert the immune system about imminent danger and to enhance the elimination of senescent cells [13,14].

Major factors contributing to cellular senescence included the activation of elevated mammalian target of rapamycin (mTOR) signaling pathway, DNA and mitochondrial damage, oxidative stress, and telomere shortening [15,16]. Moreover, inflammaging can partially result from dysfunctional or senescent T cells [[17], [18], [19]]. Recent studies suggest that senescent cells disrupt biological functions, contributing to reduced lifespan and increasing the risk of various age-related diseases, including Alzheimer's disease (AD) and Parkinson's disease (PD) [3].

The brain consumes 10 times more oxygen-dependent respiration by weight than the rest of the body for energy production [20]. The human brain contains a lot of fatty acids that are prone to peroxidation and has a higher iron content in certain areas. It also consumes 20 % of total oxygen consumption but has lower antioxidant activity than other tissues, making it more susceptible to oxidative damage than other body tissues [20]. High levels of energy supply through oxidative mitochondrial respiration generates excess oxidative stress [[21], [22], [23]]. The aging brain has increased metal concentrations in the blood-brain barrier at the junction of nerves and blood vessels, eventually leading to high levels of redox metals such as iron, zinc, and copper. This buildup of metals makes the aging brain more susceptible to the onset of neurodegeneration near nerve cells in the brain [24]. Therefore, the brain is highly susceptible to oxidative stress and subsequent damage, leading to aging and neurological damage such as AD and PD [3,25,26].

A variety of molecular mechanisms are considered crucial in determining an individual's lifespan, such as telomere attrition, mitochondrial function, genomic instability, autophagy, inflammation, and alterations in the Klotho and sirtuin signaling pathways [25,[27], [28], [29]]. Given space constraints, this review focuses specifically on the therapeutic potential of ginseng through the modulation of Treg cells and mitochondrial interventions in anti-aging treatments.

## 2 Ginseng as anti-aging remedy

A key objective of anti-aging research is to mitigate the adverse effects of aging and extend the healthy lifespan of the aging population. Given this trend, it is essential to understand the physiological changes associated with aging and develop strategies to promote healthy aging [[30], [31], [32]]. As a result, the identification of anti-aging drugs is crucial for delaying the aging process and preventing age-related diseases [13,26,[33], [34], [35]].

Ginseng possesses anti-aging, anti-apoptotic, anti-inflammatory, antioxidant, and neuroprotective and immunomodulatory properties, and has been used since ages for its life-extending outcomes [[30], [31], [32]]. Ginseng products are typically categorized into two types based on their processing methods: Panax ginseng (PG) and Red ginseng (RG). PG is produced by naturally drying raw ginseng in the sun, while RG is derived from raw ginseng through a process of steaming and prolonged drying. The main active compounds of ginseng include ginsenosides, polysaccharides, flavonoids, and polyphenols, with ginsenosides being the primary components responsible for its various pharmacological effects and significant anti-aging properties [[30], [31], [32],^34^]. Many studies suggest that ginsenosides regulates microbial activity and possess antioxidant properties by upregulating endogenous antioxidant enzymes in aging mouse models [[33], [34], [35]]. Currently, ginseng and its active ingredients are widely considered as effective treatments for age-related diseases [13,26,[33], [34], [35]]. This review summarizes the evidence supporting ginseng's anti-aging effects through various mechanisms, as outlined in Table 1.

**Table 1:** Therapeutic potential of Ginseng and related ginsenosides to attenuate age-related diseases using *in vivo* and *in vitro* models.

Ginseng (type/dosage form)	Aging model (Cells, Animal)	Aging signaling pathway and mechanism	Ref
**Cell models**			
Ginsenoside Rg1 (5, 10 and 20) μM	Human diploid fibroblasts	Ginsenoside Rg1 induced premature senescence and increased in the P16 and P21 protein levels and diminished intracellular ATP level and mitochondrial complex IV activity.	11
Ginsenoside Rg3 for 24 h	Ultraviolet-irradiated human dermal fibroblasts	Rg3 restored mitochondrial ATP to protect mitochondrial dysfunction and increased the antioxidant proteins level such as NRF2 and heme oxygenase-1.	36
Ginseng oligopeptides (GOPs) (25, 50 and 100) μg/mL, for 4 h	Endothelial cells	GOPs enhanced cell fate stability and mitochondrial biosynthesis by activating PGC-1α expression to improve life and health span.	37
Ginsenoside Rb2 (10, 40 and 80) mM	Human dermal fibroblasts	Rb2 regulated the AMPK-mTOR pathway to enhance autophagy resulting in the restoration of cellular senescence.	96
***Caenorhabditis elegans***			
Probiotic fermented ginseng (40, 80 and 160) μg/mL for 72 h	*C. elegans*	Probiotic fermented ginseng increased antioxidant enzymes such as T-SOD, GSH-PX and CAT to improved lifespan of *C. elegans*.	32
Polysaccharides extracted from ginsenoside residues (GRP)	*C. elegans*	GRP reduced levels of lipofuscin and ROS and increased SOD activity. GRP increased the abundance of Prevotellaceae and Bacteroidaceae, *Akkermansia* and *Bacteroides*.	38
*Panax notoginseng* polysaccharides main root polysaccharide (0.1 mg/mL), branch root polysaccharide (0.1 mg/mL) and fibrous root polysaccharide (0.1 mg/mL)	*C. elegans*	*Panax notoginseng* polysaccharides had higher activities of SOD and CAT, and low level of MDA in *C. elegans* suggesting increased survival rate and the lifespan extension thus displaying powerful anti-aging ability *in vivo*.	39
Ginseng volatile oil (GVO) (0, 12.5, 25 and 50) μg/mL	*C. elegans*	GVO exert its antiaging function and increased the health, lifespan, and antioxidant capacity of *C. elegans* in a dose-dependent manner.	99
Total ginsenosides (TGS) 10 g/mL for 3 days	*C. elegans*	TGS reduced lipofuscin accumulation and promoted lipid metabolism, prolonged the longevity and lifespan of *C. elegans* in response to heat and oxidative stress via the reduction of ROS.	40
Total ginsenosides (TGS) 0.2 mg/mL	*C. elegans*	TGS extended the lifespan of worms by 14.02 % by activating the signaling pathways of anti-oxidant regulation and longevity, including the NRF2/SKN-1, SIRT1/SIR2 and FOXO/DAF-16 signaling pathways.	41
***Drosophila melanogaster***			
Red ginseng (RG) (12.5, 15 and 17.5) mg/mL	*D. melanogaster*	RG significantly extended the lifespan by reducing the expression of Pebp1 and AKT pathway while up-regulating ERK in the flies.	42
KRG tonic (0.12, 1.2, and 12 μg/ml)	*D. melanogaster*	KRG prolonged lifespan, enhanced resistance to mitigate environmental stresses.	43
***In vivo* aging models**			
***Panax ginseng***			
*Panax ginseng* (ginseng protein) (0.05 and 0.1) g/kg twice daily for 30 days	D-Gal-induced- Wistar rats (Alzheimer's disease)	Ginseng protein increased Bcl-2/Bax and reduced the content of Aβ1-42 and p-tau to improve memory ability via PI3K, p-Akt/Akt signaling pathway activation in the hippocampus in aging rats.	26
*Panax ginseng* (PG) and red ginseng (RG) (800 mg/kg) for 9 weeks	Male D-Gal-induced ICR mice	RG increased *Bifidobacterium, Akkermansia, Lactobacillus, Barnesiella*, and *Prevotella*. PG and RG decreased acetylcholinesterase (AChE), and MDA levels, and improved SOD, and CAT in the serum. PG and RG inhibited NF-κB, Caspase-3 and the PI3K/Akt pathways.	34
*Panax ginseng* berry extract (GBE) and soluble whey protein hydrolysate (WPH) (700, 900 and 1100) mg/kg for 8 weeks.	Male C57BL/6J mice, 10 months old	GBE + WPH improved mitochondrial biogenesis via PI3K/Akt pathway activation in the muscle.	46
Red ginseng extract 200 mg/kg/d for 3 months	Male C57BL/6 mice, 21 months old	Red ginseng treatment attenuated memory impairment and repressed the oxidative stress markers iNOS, COX-2, TNF-α, and IL-1β expressions via the restoration of antioxidative-related enzymes Nrf2 and HO-1 in aged mice.	53
KRG water extract 200 mg/kg for 4 months.	Male SD rats, 12 months old	KRG extract attenuated lipid peroxidation and oxidative stress damage by restoring antioxidant potential including CAT, glutathione reductase, and glutathione-S-transferase, GSH-Px, SOD, including vitamins C and E deficiencies.	59
KRG 200 mg/kg/day for 30 days	Male C57BL/6J mice, 17 months old	KRG increased the expression of GDF-11, Lin28a, SIRT1, splenic Treg cells and IFN-γ expressing NK cells in the aged mice.	60
Black ginseng 300 mg/kg for 4 weeks	Male C57BL/6 mice, 18 months old	Black ginseng supplementation attenuated cellular senescence and downregulated age-related inflammatory genes via p53, p21 and p16 activation in liver, skeletal muscle and white adipose tissues.	14
Fecal microbiota transplantation treated with ginseng-supplemented	Male C57BL/6N mice, 20 months old	FMT treated with ginseng-supplemented increased the abundance of beneficial bacteria of *Bacteroides*, *Dubosiella*, Lachnospiraceae, and improved the proportion of Treg cells in the fecal samples of natural aged mice.	30
Ginseng water decoction (0.9, 1.8 and 3.6) g/kg	Male D-Gal-induced ICR mice, 6 weeks old	Ginseng delayed Lgr5 and Olfm4 expression and increased SCFA, such as acetic acid, butyric acid, and propionic acid and enhanced intestinal function by activating the Wnt/β-catenin signaling pathway.	98
***Panax notoginseng***			
*Panax notoginseng* saponins (PNS) (10, 30 and 60) mg/kg/day for 6 months	Male SD rats, 24 months old	PNS significantly decreases cardiomyocyte apoptosis and increased Mn-SOD, PGC-1α, LC3β, and Beclin-1 levels to improve age-associated mitochondrial dysfunction in a dose-dependent manner.	93
***Panax quinquefolium***			
*Panax quinquefolium* (0.5 and 2.5) g/kg	Female Fischer rats, 4 months old	Ginseng supplementation in rats decreased age-related oxidative damage in the heart and oxidative muscle fibers.	45
Garden ginseng (GG) and ginseng under forest (FG) GG and FG (400 mg/kg/day) for 6 weeks	Male D-Gal-induced ICR mice, 8 weeks old	GG and FG enhanced hippocampal lesions by modulating apoptosis-related proteins, PI3K/AKT/mTOR pathway, and SIRT1/NF-κB pathway in the aging brain. GG and FG increased the relative abundance of beneficial *Lactobacillus* bacteria via the microbiota-gut-brain axis.	9
Maltol (50 and 100) mg/kg per day, for 4-weeks	Male D-Gal-induced ICR mice, 8 weeks old	Maltol activated aging-associated proteins including p53, p21, and p16, PI3K/Akt signaling pathway followed by inhibiting oxidative stress factors MDA, and increasing the levels of antioxidant enzymes in liver and kidney cell senescence and injury.	55
Maltol, produced from heat treatment of ginseng, (50 and 100) mg/kg per day by oral gavage	Male D-Gal-induced ICR mice, 8-weeks old	Maltol treatment decreased ROS and MDA production via activation of the PI3K/Akt-mediated Nrf2/HO-1 signaling pathway in aging brain.	57
**Ginsenosides**			
Ginsenoside (0.028, 0.05, and 0.112) % (w/v) for 8 months	Female C57BL/6J mice, 12 months old	Ginsenoside prevented memory impairment and markedly increases SOD, GSH-Px, and thiobarbituric acid reactive substances (TBARS) in the hippocampus.	51
Ginsenoside Rb1 every 3 days, starting from the age of 1 year until 2 years of age	C57BL/6J mice, 24 months old	Ginsenoside Rb1 alleviated markers of oxidative stress, such as NO, MDA and NADPH, and by the increased ROS-scavenger GSH contents to treat neurological disorders.	44
Ginsenoside Rb1 or Re 10 mg/kg/day for consecutive 35 days	GPx-1 KO mice 16-month-old	Ginsenosides Rb1 or Re ameliorated aging-associated memory impairments in the absence of GPx-1 by downregulating the initial oxidative damage followed by PAFR, NFκB, microglial, and ERK activation.	84
Ginsenoside Rg1 (10 and 20) mg/kg	Male D-Gal-induced Kunming mice (3–4 weeks old)	Rg1 attenuated neuronal apoptosis and prevent cognitive deficits via activation of FGF2-Akt and BDNF-TrkB signaling pathways in the hippocampus.	85
Ginsenoside Rg1 20 mg/kg/day, daily for 28 days	D-Gal-induced C57BL/6 mice, 6–8 weeks old.	Rg1 improved cognitive impairment by attenuating senescence of neural stem cells. Rg1 increased antioxidant activity of SOD and GSH-Px in aged mice. Rg1 furthermore reduced the Akt/mTOR pathway.	15
Ginsenoside Rg1, 6 mg/kg every third day for 24 months	Female C57BL/6J mice, 12 months old	Activate mTOR pathway and synaptic plasticity-associated proteins in hippocampus.	86
Ginsenoside Rg1 (5 and 10) mg/kg for 9 weeks	SAMP8 mice, 6 months old	Rg1 attenuated aging-associated liver injury and fibrosis and reduced levels of ROS, IL-1β, and NOX4, NF-κB, caspase-1, NLRP3 inflammasomes in SAMP8 mice.	47
Ginsenoside Rg1 (2.5, 5.0 and 10) mg/kg for 3 months	Male SAMP8 mice, 6 months old	Ginsenoside Rg1 improved the cognitive function from the upregulation of BDNF protein via PKA/CREB pathway in SAMP8 mice.	82
Ginsenoside Rg1 20 mg/kg/d for 4 weeks	Male D-Gal-induced SD rats, 6–8 weeks old	Ginsenoside Rg1 decreased ROS, MDA, GSH while IL-2, IL-6, granulocyte-macrophage colony stimulating factor (GM-CSF), TNF-α, SOD, and the proliferative capacity of splenocytes and thymocytes were increased in D-galactose-induced aging rats.	49
Ginsenoside Rg1 20 mg/kg/d for 4 weeks	Male D-Gal-induced SD rats	Rg1 increased GSH-Px, SOD, SOX-2 level, and reduced IL-1b, IL-6 and TNF-a level and downregulated the expression of hippocampal cellular senescence associated genes p53, p21 Cip1/Waf1 and p19 Arf in the hippocampus of aged rats.	7
Ginsenoside Rg2 (10 and 20) mg/kg for 4 weeks	Male D-Gal-induced ICR mice, 8 weeks old	Rg2 restored impaired memory function, choline dysfunction, and redox system imbalance and decreased the over-expression of aging-related proteins such as p53/p21/p16ink4α to maintain mitochondrial function in brain.	28
20(R)-ginsenoside Rg3 (10 and 20) mg/kg/day for 4 weeks	Male D-Gal-induced ICR mice	20(R)-Rg3 markedly increased Bcl-2, CAT and SOD, and decreased Bax gene via PI3K/AKT signaling pathway activation in liver and kidney.	48
Ginsenoside Re 10 mg/kg/day for 30 consecutive days	Klotho deficient mice, 14 months old	Ginsenoside Re enhanced memory impairments and attenuated the ROS, NOX activity via upregulation of GSH-Px mediated by Nrf2/GPx-1/ERK/CREB signaling in aged Klotho deficient mice.	27
20(S)-protopanaxatriol (10 and 20) mg/kg for 4 weeks	Male D-Gal-induced ICR mice, 8 weeks old	20(S)-protopanaxatriol increased TFEB/LAMP2 to enhance mitochondrial autophagic flow to delay brain aging.	29
Ginsenoside Rh2, 200 nM	H_2_O_2_-induced aging porcine oocytes	Rh2 supplementation enhanced mitochondrial biogenesis-related genes SIRT1 and PGC-1α, and the antioxidant gene SOD1 in H_2_O_2_-induced aging porcine oocytes.	90
Ginsenoside Rh4 (50, 100, and 200 mg/kg) for 6 weeks	Male D-Gal-induced C57BL/6 J mice 4–6 weeks old	Ginsenoside Rh4 enhanced mitochondrial homeostasis, and delayed skeletal muscle aging by regulating the PGC-1α-TFAM and HIF-1α-c-Myc pathways via activating SIRT1.	94
American ginseng saponin Rb1 and Re. Rb1 (30 mg/kg), Re 15 mg/kg, and Rb1 + Re (30 mg/kg + 15 mg/kg (co-intervention).	Male C57BL/6 mice, 18 months old	Rb1 and Re delayed the decline of the immune system and reduced the IL-2, IL-6, IL-17, INF and TNF in aged mice.	54
**Saponins fraction**			
SPJ (10 and 60) mg/kg/d for 6 months	SD rats, 18 months old	SPJ improved age-associated renal fibrosis by inhibiting TGF-β1/Smad, NFkB signaling pathways and activating Nrf2-ARE signaling pathways	10
SPJ (50, 100 and 200) mg/kg	Male D-Gal-induced Wistar rats	SPJ decreased lipofuscin and ROS accumulation levels and increased Nrf2, and SIRT1, Mn-SOD, HO-1 to improve memory impairment in aging brain.	52
SPJ (10 and 60) mg/kg/day.	Male SD rats, 18 months old	SPJ significantly inhibited Bax, IL-1β, TNF-α and NF-κB p65, and increase the expression of Bcl-2, Bcl-2/Bax, p-AMPK/AMPK and Sirt1 in the cardiac tissues of natural aging rats.	8
SPJ (10 and 30) mg/kg for 4 months	Male D-Gal-induced-SD rats, 18 months old	SPJ improved the mitochondrial morphology and neuronal degeneration via upregulation of mitofusin and optic atrophy 1 proteins while decreasing dynamin-like protein in the hippocampus of aging rats.	21
SPJ (10 and 30) mg/kg for 6 months	Male SD rats, 24 months old	SPJ improved the cardiac aging phenotype by activating AMPK activity, which then inhibits mTOR activity ultimately leading to autophagy activation.	16
SPJ (10, 30 and 60) mg/kg once daily	SD rats, 18 months old	SPJ conferred neuroprotection and increased the activities of SOD, GSH-Px, LC3II, SIRT1 protein whereas decreased MDA, PGC-1α contents in the hippocampus of aging rats.	23
**Non-saponin fraction**			
Non-saponin fraction with rich polysaccharide (NFP) 150 mg/kg for 8 weeks.	Male and female 5xFAD transgenic mouse model of Alzheimer's disease	NFP treatment ameliorated mitochondrial deficits in Aβ-treated HT22 cells. NFP increased cell proliferation, alleviated the accumulation of Aβ, neuronal loss, and mitochondrial dysfunction.	35
**Miscellaneous**			
Arginyl-fructosyl-glucose (40 and 80) mg/kg for 4 weeks	Male D-Gal-induced ICR mice, 8 weeks old	Arginyl-fructosyl-glucose attenuated mitochondrial dysfunction and ROS activity to delay brain aging.	25
Gintonin (50 and 100 mg/kg/day, for 4 weeks	Male D-Gal-induced C57BL/6 mice, 5 weeks old	Gintonin attenuated hippocampal aging, resulting in the improvement of cognitive functions and brain aging.	56

**Abbreviations**; Aβ, Amyloid β; AD, Alzheimer's disease; AHN, Adult hippocampal neurogenesis; Akt, protein kinase B; AMPK, Adenosine monophosphate-activated protein kinase; ATP, Adenosine Triphosphate; *C. elegans*, *Caenorhabditis elegans*; CAT, Catalase; CD4, cluster of differentiation; CRP, C-reactive protein; D-Gal, D-galactose; ETC, Electron transport chain; FMT, Fecal microbiota transplantation; FoxO3a, Forkhead Box Transcription Factor; FOXP3, forkhead box P3; FG, Ginseng under forest; GG, Garden ginseng; GOPs, Ginseng oligopeptides; GRP, Polysaccharides from ginsenoside residues; GSH-Px, Glutathione peroxidases; GSH, glutathione; H_2_O_2_, Hydrogen peroxide; IFN-γ, Interferon-gamma, IL, Interleukin; iNOS, Inducible nitric oxide synthase; KRG, Korean red ginseng; MDA, Malondialdehyde; MAPK, Mitogen-activated protein kinase; MDSC, Myeloid-derived suppressor cells; mtDNA, Mitochondrial DNA; Mn-SOD, Manganese superoxide dismutase; mTOR, Mammalian target of rapamycin; NF-κB, Nuclear factor-κB; NFP, Non-saponin fraction with rich polysaccharides; NK, Natural killer; NLRP, Nucleotide-binding oligomerization domain, Leucine rich Repeat and Pyrin domain containing; NOX, NADPH Oxidase; Nrf2, Nuclear factor-erythroid 2-related factor 2; PD, Parkinson's disease; PG, *Panax ginseng*; PGC-1α, Peroxisome proliferator-activated receptor-γ coactivator-1α; PI3K, Phosphatidylinositol-3-kinase; PNS, *Panax notoginseng* saponins; PMN, Polymorphonuclear; PPARγ, Peroxisome proliferator-activated receptor gamma, ROS, Reactive oxygen species; RG, Red ginseng; SAMP8, Senescence-accelerated P8 mice; SOD; Superoxide dismutase; SD, Sprague-Dawley, SPJ, Saponins of *Panax japonicus*; TGF-β, Transforming growth factor-β; Th, T helper; TNF-α, Tumor necrosis factor alpha; Treg, regulatory T cell.

Ginseng's anti-aging properties have been extensively studied using *in vitro* and *in vivo* models, with findings indicating its ability to inhibit aging markers and extend lifespan in human fibroblasts [11,12,36,37], *Caenorhabditis* (*C*)*. elegans* [32,[38], [39], [40], [41]], *Drosophila* (*D*)*. melanogaster* [42,43], and mice or rat [8,15,16,21,26,30,34,35,[44], [45], [46], [47], [48], [49]] (Fig. 2 and Table 1). However, studies have also reported that Korean and American ginseng are ineffective in promoting longevity and protecting against cold-water stress [50].

### 2.1 Ginseng, aging and oxidative stress

The pathological mechanisms underlying aging are intricate, varied, and often debated. Among the prominent theories, the free radical theory suggests that the damage caused by oxygen free radicals is a widely accepted explanation for aging [7,34,51]. This theory hypothesizes that sustained disruption caused by reactive oxygen species (ROS), a key mediator of oxidative stress, progressively increases throughout the natural aging process. It is well established that elevated oxidative stress can result in cellular damage, compromising DNA, phospholipids, and proteins involved in inflammatory damage. This ultimately accelerates aging and leads to organ dysfunction, cognitive decline, and age-related degenerative diseases [15,23,44,52]. Oxidative stress may activate NF-κB transcription factors, which can stimulate the release of pro-inflammatory cytokines during the entire aging process, further intensifying oxidative damage and apoptosis [[8], [9], [10]]. ROS homeostasis is regulated by antioxidant enzymes within the body. An effective therapeutic approach for specific aging processes underscores the need for natural supplements to provide potent and safe ROS scavengers, thereby improving healthy aging and reducing the prevalence of age-related disease [44].

Ginseng and its active components are known for their strong antioxidant properties and anti-aging effects [23,52]. Ginsenosides have been linked to the inhibition of the NF-κB signaling pathway, which is typically regulated by inflammatory cytokines such as TNF-α, IL-1, IL-6, and by enzymes like iNOS and COX-2 [7,53]. Pretreatment with Rb1 or Re, the levels of pro-inflammatory cytokines TNF, INF, IL-2, IL-6, and IL-17 were significantly reduced in aged mice [54]. Red ginseng treatment also suppressed the production of oxidative stress markers COX-2, IL-1β, iNOS, and TNF-α by restoring antioxidant-related enzymes nuclear factor erythroid 2-related factor 2 (Nrf2) and heme oxygenase-1 (HO-1) in aged mice [53]. The phosphatidylinositol-3-kinase (PI3K)/protein kinase B (Akt) pathway is a crucial antioxidant transcription factor involved in aging, and significant efforts have been made to explore its role in antioxidants and aging [26,48,55]. Ginseng has been extensively researched for its ability to regulate the PI3K/Akt pathway. Moreover, the inhibition of the PI3K/Akt signaling pathway has been linked to a reduction in brain damage during the aging process [26,42,43,55]. Ginseng proteins have demonstrated memory-enhancing and neuroprotective effects in AD rats via the PI3K/Akt and Bax/Bcl-2 pathways [26]. In fact, RG has been shown to significantly extend the lifespan of *D. melanogaster* by upregulating ERK and inhibiting the Akt pathway [42,43] (Fig. 2 and Table 1). Total ginsenosides (TGS), including Rg1, Re, and Rb1, have been found to extend both lifespan and health span by enhancing antioxidant activity and maintaining mitochondrial homeostasis. This is primarily achieved through the regulation of SKN-1, SIR 2.1, and DAF-16 signaling pathways [41]. In the heart tissue of naturally aged rats, saponins from *Panax japonicus* (SPJ) effectively inhibited cardiomyocyte apoptosis and inflammatory cell infiltration by suppressing the protein expression of Bax, IL-1β, TNF-α, and Ac-NF-κB p65, while increasing the expression of Bcl-2, Bcl-2/Bax, p-AMPK/AMPK, and SIRT1 [8]. Additionally, SPJ has been shown to improve the cardiac aging phenotype in naturally aging rats, primarily by activating AMPK activity [16] (Fig. 2 and Table 1).

D-galactose (D-Gal) is widely recognized as an age-accelerating agent as high-dose intake of D-Gal for long-term can lead to excessive production and accumulation of ROS *in vivo*, causing oxidative stress and organ damage that mirrors the symptoms of natural aging [7,26,34,55]. Liver and kidney dysfunction, which are closely linked to aging, are particularly vulnerable to the oxidative damage induced by D-Gal [47,48,55]. Numerous studies have explored the aging mechanisms in the brain, liver, and kidney using established D-Gal animal models [28,47,48,55]. Gintonin extract from ginseng has been shown to alleviate D-Gal-induced brain aging-related dysfunctions, improving cognitive functions [56]. Data showed that PG and RG exhibited anti-inflammatory properties by inhibiting NF-κB translocation and the phosphorylation of PI3K/Akt in the D-Gal-induced aging brain [34]. Treatment with Garden ginseng (GG) and ginseng under forest (FG) mitigated D-Gal-induced oxidative stress and improved cognitive and spatial learning impairments, neuronal damage, and apoptosis in aging mice [9,33]. Furthermore, ginsenoside Rg1 alleviates neural stem cell senescence and cognitive dysfunction in aging mice by reducing oxidative stress [15]. In D-Gal-induced aging rats, the ginsenoside Rg1 significantly reduced ROS, malondialdehyde (MDA), glutathione (GSH), and the expression of senescence-associated proteins in the spleen and thymus [49]. Additionally, Rg3 effectively alleviated D-Gal-induced apoptosis in the kidney and liver through the PI3K/AKT signaling pathway [48]. Treatment with SPJs improved age-related renal fibrosis by downregulating inflammatory NF-kB, TGF-β1/Smad signaling pathways, and activating Nrf2-ARE pathways in aging mice [10] (Fig. 2 and Table 1).

Maltol, a natural compound (3-hydroxy-2-methyl-4-pyrone), is produced during the heat treatment of ginseng (*P. ginseng* C.A Meyer) and other traditional Chinese medicines. It has been shown to slow down age-related brain aging and reduce behavioral dysfunction and neurological deficits [57]. Maltol supplementation improved cellular senescence and damage caused by D-Gal-induced accelerated aging, suggesting its potential therapeutic benefits for age-related liver and kidney damage [55]. Maltol supplementation could alleviate D-Gal-induced aging and injury in the liver and kidney by reducing oxidative stress through the activation of the p53/p21/p16 and PI3K/Akt signaling pathways, rendering this as a promising agent for future research on therapeutic strategies to treat or prevent age-related liver and kidney diseases [55]. Black ginseng supplementation reduced the activation of the canonical senescence pathway, including p53-dependent p21 and p16, in various metabolic organs such as the liver, skeletal muscle, and white adipose tissue of aged mice [14]. It is well established that p53 and p21 are crucial for regulating the cell cycle, DNA repair, apoptosis, and other biological processes, while p16 functions as a regulator of cell senescence and can lower the risk of aging-related diseases, particularly those linked to liver and kidney dysfunction [47,48,55]. Moreover, p53 activation leads to senescence through several downstream targets, while upregulation of p21 by p53 can result in cell cycle arrest [14,28,55]. In mice treated with D-Gal, the overexpression of senescence-associated signals such as p53, p21, and p16 in the liver and kidney was reversed by maltol treatment [55]. Additionally, PI3K/Akt signaling plays a complex role in inflammatory pathways. Previous studies suggest that PI3K and Akt proteins are involved in the phosphorylation and nuclear translocation of NF-κB p65 by interacting with upstream proteins [58].

Consistent with these observations, the reduced expression of p-IκB α, NF-κB, and p53 in PG- and RG-treated mice attenuated D-Gal-induced inflammatory response, thereby enhancing antioxidant activity. It is conceivable that RG and PG might help to prevent brain aging by regulating apoptosis-related protein expression (Bcl-2, Caspase-3, and Bax) to varying degrees manifested by PI3K/Akt and NF-κB-mediated anti-apoptotic pathways [34]. D-Gal administration significantly reduced Bcl-2 expression, while increasing the levels of Bax and caspase-3, leading to higher apoptosis in brain tissues. In contrast, GG and FG treatments protected cell proliferation and tissue remodeling by increasing Bcl-2 expression, reducing caspase-3 and Bax expression, and modulating apoptosis-related proteins, the PI3K/AKT/mTOR pathway, and the SIRT1/NF-κB pathway [9] (Fig. 2 and Table 1). The Nrf2 and phosphoinositide 3-kinase (PI3K)/Akt pathways are crucial in the transcription of antioxidant factors, mediating the expression of enzymes like catalase (CAT), GSH, superoxide dismutase (SOD), and other cytoprotective proteins involved in the removal of oxygen radicals [23,34,57].

The antioxidant system network is a critical factor in the anti-aging process. In D-Gal-induced aging, the activities of vital endogenous antioxidant enzymes such as SOD and CAT are diminished, which leads to reduced organ function. Therefore, clearing or preventing ROS formation is essential for mitigating D-Gal-induced senescence [26,34,49,55,59].

The anti-aging effects of ginseng components related to suppression of oxidative mechanism have been extensively documented. For instance, ginsenoside Rg1 enhanced the activity of antioxidant enzymes like SOD and glutathione peroxidase (GSH-Px), effectively neutralizing accumulated free radicals and alleviating oxidative damage, thereby delaying aging [7,26,38,39]. Administration of ginsenosides in mice and rats has been shown to decrease lipid peroxidation, eliminate free radicals, and boost the activities of CAT, glutathione reductase, and GSH-Px [7,27]. *P. ginseng* reduced lipid peroxidation and restored antioxidant potential, including CAT, SOD, GSH-Px, glutathione reductase, and glutathione-S-transferase, by attenuating oxidative stress in aged rats [59]. In D-Gal-induced mice, Rg3 increased antioxidant levels of CAT and SOD, whereas a decrease in lipid peroxidation products such as MDA and 4-hydroxynonenal levels [48]. Similarly, maltol treatment remarkably reduced MDA levels and increased CAT and SOD levels in D-Gal-induced accelerated senescence [57].

### 2.2 Ginseng, aging and immune/inflammatory disorders

**Fig. 1:**
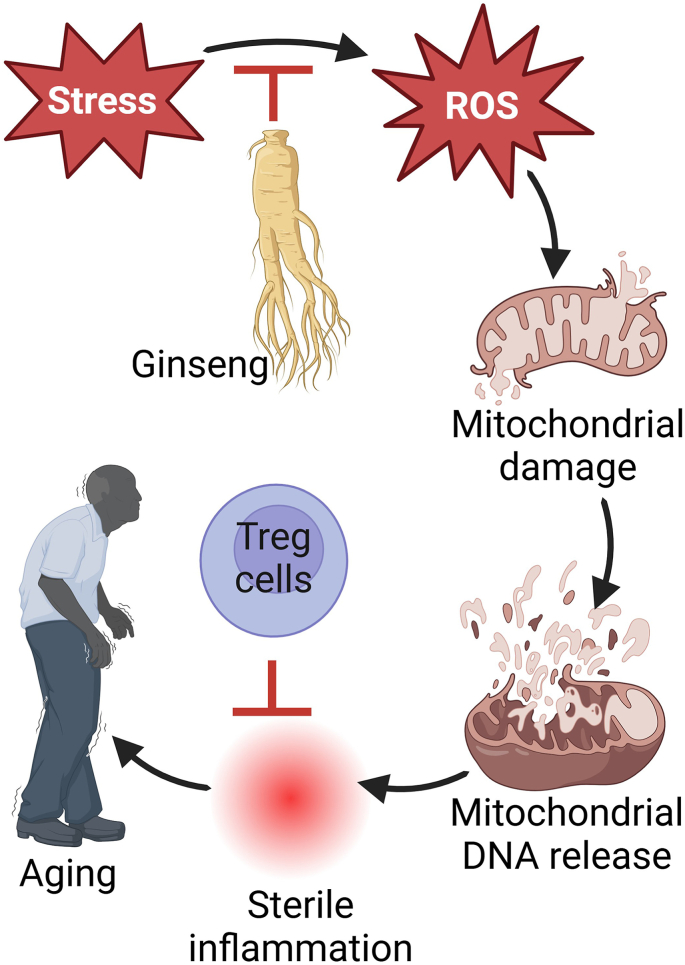
**Therapeutic potential of ginseng on aging-related inflammaging.** Stress perpetuated by inflammatory mediators and genetic factors induced ROS production and increased level of proinflammatory cytokines leading to mitochondrial DNA damage and inflammatory phenotypes. This accelerates the progression and cascade of aging process. Ginseng and its active components, ginsenosides suppress the aging-induced oxidative stress via the antioxidant potential and anti-inflammatory Treg cells activation leading to the restoration of mitochondrial biogenesis and function (image created using the BioRender application).

As the functions and compliance of the immune system and physiological processes gradually decline with age, thus accelerating cellular aging and leading to the onset of age-related diseases [54,60]. A key mechanism in the phenomenon of inflammaging is the dysregulation of immune cells, driven by elevated levels of inflammatory cytokines [4,6,17]. This process is manifested by structural changes in the immune system, collectively referred to as “immunosenescence” [5,6,61]. Mitochondria generate energy through the electron transport chain (ETC), utilizing oxygen as the final electron acceptor. Recent studies have emphasized mitochondria as crucial regulators of innate immunity, with mitochondrial DNA (mtDNA) being released into the cytoplasm, extracellular space, or bloodstream to activate multiple innate immune signaling pathways [62]. Aging and prevalent diseases in the elderly have been linked to somatic mutations that disrupt ETC function. As mitochondrial function declines with age, mtDNA is shed under apoptotic stress, leading to persistent low-grade sterile inflammation that triggers the aging process. Once mtDNA is released into the extracellular space, it acts as a damage-associated molecular pattern (DAMP), interacting with danger signal receptors and initiating an innate immune inflammatory response (sterile inflammation) (Fig. 1) [20,63]. The increased presence of pro-inflammatory factors subsequently induces the expression of anti-inflammatory factors to maintain homeostasis. The age-related activation of the immunosuppressive network is characterized by an increase in the numbers of macrophages (Mreg/M2c), myeloid-derived suppressor cells (MDSC), regulatory T (Treg) cells, and anti-inflammatory cytokines such as IL-10 and TGF-β. This network also induces enzymes like arginase 1 (Arg1), which hydrolyzes arginine into ornithine and urea, leading to arginine depletion, and indoleamine 2,3-dioxygenase (IDO), which converts L-tryptophan to N-formylkinurenine, resulting in further immunosuppression. It is well-established that MDSC and Treg cells can reduce the cytotoxicity of natural killer (NK) cells [5,61,64,65], thus preventing the clearance of cellular senescence from aging organs [17].

**Fig. 2:**
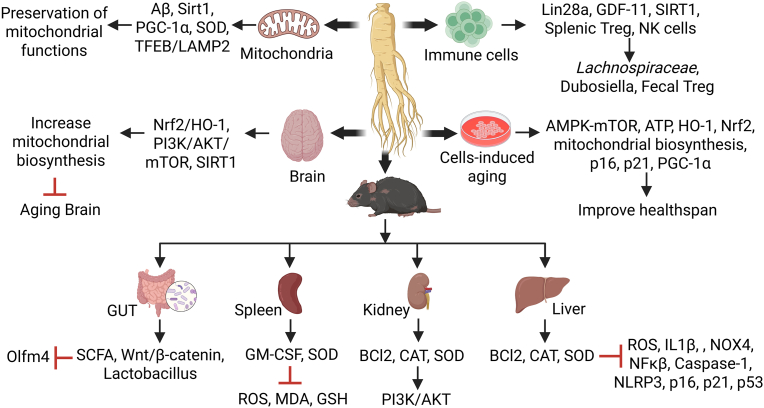
**Signaling pathways and possible mechanism underlying anti-aging potential of ginseng and related ginsenosides.** Aging-induced oxidative stress and mitochondrial damage responsible for various age-related pathologies are highlighted using *in vitro* and *in vivo* models. This oxidative damage induces the proinflammatory phenotypes into tissues resulting organs dysfunction and accelerate aging process. Ginseng supplements attenuated resultant inflammaging and improved organs function by mitigating mitochondrial dysfunction, and oxidative stress markers through various mechanisms, including the regulation of gut microbiota and modulation of age-related gene expression. This anti-aging potential of ginseng is primarily achieved via the activation of Treg cells expression, and the restoration of mitochondrial biogenesis by regulating antioxidant genes expression mechanism thus contributing to a healthy lifespan (image created using the BioRender application).

Redox status regulates T cell differentiation and activity, which in turn influences epigenetic modifications, transcription, and the stability of forkhead box P3 (FOXP3), a pivotal transcription factor that governs the development and function of Treg cells. Age-related oxidative stress has been linked to an increase in both the number and function of Treg cells [66,67]. Treg cells are typically characterized by the expression of FOXP3^+^ and cluster of differentiation (CD4)^+^ markers with emphasis to promote tissue regeneration and repair, regulate cell differentiation and proliferation, and maintain immune homeostasis [68,69]. These cells form part of an immunosuppressive network that helps sustain immune tolerance and prevent autoimmune responses. However, it is well documented that Treg cell function declines with age, which has led to the exploration of Treg cells-targeted immunotherapy as a potential treatment for aging, autoimmune diseases, and cancer. Tissue-resident Treg cell populations accumulate from birth until adulthood, and then abruptly decline in the elderly [70]. Although the amount of peripheral Treg cells in older mice increased, the functional activity of Treg cells was significantly reduced compared to younger mice [71]. Moreover, aged mouse Tregs senesce preferentially over young mouse Tregs. Treg cells age and decline in function more commonly than conventional T (Tconv) cells. Treg cells from aged mice inhibited Tconv function at a lower efficiency than in young Treg cells. Aged mouse Treg cells show deteriorated Treg function compared to young mouse Treg cells thus aged Tregs could not protect inflammation efficiently than young Tregs. In addition, preferentially in aged Tregs, ROS-related programs were upregulated. They also showed that Treg senescence could be suppressed by DCAF1 [72]. Therefore, gaining an insight into the Treg cells mechanism could lead to the development of novel therapies for age-related disorders [73]. Anti-inflammatory Treg cells have demonstrated neuroprotective effects [30,74,75], inhibiting age-related inflammation and promoting retinal regeneration [75], as well as addressing autoimmune diseases like vasculitis [76]. In patients with giant cell arteritis, the loss of Treg cells contributes to the progression of granulomatous vasculitis [76].

Aging is also associated with an increase in MDSCs in the circulation of both mice and humans [5,61]. This age-related increase in MDSC numbers is evident in frail elderly individuals (67–99 years) and community-dwelling seniors (61–76 years), who exhibit significantly higher levels of polymorphonuclear (PMN)-MDSCs in circulation compared to healthy adults (19–59 years) [65]. The elevated presence of MDSCs with age is largely driven by the activation of NF-κB signaling in the spleen and bone marrow [64]. Additionally, MDSCs isolated from the lymphoid organs in aged mice significantly attenuated antigen-induced T cell proliferation, indicating that the expansion of immunosuppressive cells with age impairs T cell function [61,64,77]. Among age-related factors, T cells orchestrate inflammaging mainly by elevated level of inflammatory cytokines and immune cell dysfunction [17]. T cells primarily consist of CD4^+^ and CD8^+^ populations. CD4^+^ T cells can further be divided into subsets, including T helper (Th)1, Th2, Th17, and Treg cells [68,69]. Among CD4^+^ T cells, Th17 cells in centenarians exhibit a senescent pro-inflammatory phenotype, markedly represented by IL-17 and ROR-γt expressions [4,18,19,74].

A novel mechanism for anti-aging research in centenarians has been proposed based on peripheral blood mononuclear cells. This research suggests that increased Th17 levels are associated with reduced secretion of pro-inflammatory cytokines in *ex vivo* differentiation assays, while a decrease in Treg cells results in the secretion of more anti-inflammatory cytokines. These findings imply that centenarians may reduce inflammaging by lowering the Th17/Treg cell ratio rendering them into anti-inflammatory secretory phenotype [4]. Moreover, an increased expansion of age-associated cytotoxic CD4^+^ T cells has been identified in human supercentenarians and tend to increase with aging [6,69]. Korean red ginseng (KRG) as an anti-aging agent modulated immune cells population and enhanced the expression of age-related genes, such as Lin28a, GDF-11, SIRT1, and increased the number of splenic Treg cells and IFN-γ-expressing natural killer (NK) cells in aged mice [60]. In a similar context, American ginseng saponins Rb1 and Re have been found to improve immune function in aging models through the suppression of pro-inflammatory mediators [54]. Treg cells expression tend to increase with aging in the blood, lymphoid, and visceral adipose tissues in human and mice [67,78]. Fecal microbiota transplantation (FMT) in ginseng-supplemented aged mice improved the proportion of Treg cells in brain tissue [30] (Fig. 2 and Table 1). However, while KRG enhanced the Treg cells population and the number of IFN-γ-expressing NK cells in the spleens of aged mice, it did not affect serum levels of Treg cell-specific cytokines such as IL-10 and TGF-β [60]. In contrast, KRG treatment did not show any comparison in splenic F4/80^+^ macrophage population between young and old mice [60]. These results underscore that KRG increases the Treg cell population in the spleen, where T cells are activated, and enhances the expression of aging-related genes in the thymocytes of older mice [60]. A significant decrease in the frequency Treg cells and an increase in Th17 cells were observed in aged participants (ages 65–80 years) compared to young adults [74]. Similarly, the expression of FOXP3 was significantly increased in elderly people of above 70 years compare to those under 30 years of age [67]. Ginsenosides exert anti-inflammatory effects by downregulating type 1 macrophages, a hallmark of inflammation. Specifically, ginsenosides Rb1, compound K, Rg1, Rg3, and Rh2 were found to shift the expression of M1 inflammatory macrophages to the M2 anti-inflammatory phenotype [79,80].

### 2.3 Ginseng, aging and neurological disorders

The brain being the most vulnerable organ to aging is highly susceptible to autophagy dysfunction, DNA damage, and oxidative stress [23,25,28]. The ability of the brain to repair nervous system gradually declines with age [35]. Age-related alterations can accelerate the onset and progression of neurodegenerative diseases, such as AD and PD [3]. These pathologies are also closely correlated to the hallmarks of aging and are characterized by the deposition of amyloid-β (Aβ) peptides, genomic alterations and instability, and cellular senescence [35] leading to the abnormal accumulation of protein aggregates, cognitive decline, and mitochondrial dysfunction in the central nervous system [81,82]. Notably, Aβ pathology is associated with neuroinflammation and neuronal cell death in AD patients [26,83]. Several studies have suggested that ginsenosides, derived from red ginseng, may offer therapeutic potential for treating age-related neurodegenerative diseases [81,83].

Aging contributes to various levels of brain damage, particularly affecting the cholinergic system and hippocampal function, which are directly linked to cognitive and memory deficits [28]. The generation of ROS and free radicals, which induces oxidative stress, can damage the hippocampus and increase vulnerability to learning and memory impairments [51]. Ginseng supplementation, such as SPJ, has been shown to improve spatial learning and memory deficits, restoring anti-apoptotic and anti-oxidative features in D-Gal-induced rats [52]. Ginsenoside Re significantly reduced ROS and NADPH oxidase (NOX) levels and activated Nrf2-mediated pathways in aged Klotho-deficient mice, enhancing memory function by upregulating Nrf2/GPx-1/ERK/CREB signaling [27] (Fig. 2 and Table 1). Long-term intake of ginsenoside treatment prevented memory impairment in aged mice by upregulating plasticity-related proteins, such as T-SOD and GSH-Px, while reducing levels of thiobarbituric acid reactive substances (TBARS) in the hippocampus [51]. The non-saponin fraction (NFP) of ginseng, rich in polysaccharides, has been shown to mitigate AD-related pathologies, including Aβ deposition, mitochondrial dysfunction, neuroinflammation, neuronal death, and impairments in adult hippocampal neurogenesis (AHN) in aged brains both *in vivo* and *in vitro* [35]. Consistently, ginsenoside Re attenuated memory impairments via the regulation of the angiotensin II AT1 receptor, GPx-1, NF-kB, Nrf2, and PAFR, demonstrating anti-aging effects [27,84]. Ginsenoside Rg1 in the hippocampus of D-Gal-induced mice inhibited neuronal apoptosis and improved cognitive deficits via activation of FGF2-Akt and BDNF-TrkB signaling pathways [85]. Ginsenoside Rb1 reduced oxidative stress markers such as NO, MDA, and NADPH by increasing ROS-scavenger GSH contents in aged mice, suggesting its potent antioxidant potential for treating age-related neurological disorders [44] (Fig. 2 and Table 1).

Gintonin attenuated D-Gal-induced hippocampal nerve cell damage and cognitive dysfunction via inhibition of ROS activity and inflammatory cytokines thereby delaying brain aging [56]. Ginsenosides Rg1 significantly improved cognitive impairment in D-Gal-induced alteration in the hippocampus, and downregulate age-related markers such as p53, p21 Cip1/Waf1 and p19 Arf [7] (Fig. 2 and Table 1). Similarly, Rg1 alleviated oxidative stress and Akt signaling by enhancing the expression of antioxidant enzymes like GSH-Px and SOD in D-Gal-induced mice [15]. Furthermore, Rg1 improved cognitive performance by inhibiting β-amyloid protein expression and activating the PKA/CREB pathway in senescence-accelerated P8 (SAMP8) mice [82]. Rg1 also significantly downregulated the expression of collagen IV, IL-1β, TGF-β1, ROS, NOX4, and NLRP3 inflammasomes in SAMP8 mice, indicating its potential to prevent aging-induced organ damage and fibrosis [47]. Moreover, prolonged administration of Rg1 in aged mice improved cognitive function via the mTOR pathway activation [86], suggesting the ability of the ginseng to inhibit neuronal stem cells aging by regulating oxidative defense mechanisms and Akt/mTOR signaling pathway [9,15].

### 2.4 Ginseng, aging and mitochondrial dysfunction

Under apoptotic stress, the efflux of mtDNA through mitochondrial outer membrane permeabilization triggers the senescence program. Additionally, other pathways, such as pyroptosis, can induce sterile inflammation (Fig. 2). This process, triggered by mtDNA release, acts as a caspase-dependent systemic inflammatory mediator that contributes to aging [22,28,63]. As such, mutations in mtDNA are linked to various genetic disorders and are closely associated to age-related diseases including aging [62]. Mitochondrial dysfunction has a major impact on aging because mitochondria regulate calcium levels, cellular energy, and oxidative balance. Mitochondrial autophagy (mitophagy) is critical for maintaining mitochondrial integrity as it contributes to protecting mtDNA damage, maintaining cellular homeostasis, and preventing ROS production. However, when calcium uptake or oxidative stress increases, mitochondrial permeability and membrane potential are disrupted, triggering a cascade of apoptotic responses. Consequently, mitochondrial dysfunction is associated with age-related degenerative symptoms [25,28,29,87]. The frequency of mtDNA point mutations and deletions increases with age, but by late life, a threshold level is reached where mitochondrial signaling and function are likely impaired [63]. Most eukaryotic cellular organisms have hundreds of copies of mDNA and exist in more than one type (heteroplasmy) due to mutations. These mutations can eventually lead to heterogeneity and dysregulation of key metabolites due to failures in signal transduction pathways that regulate mitochondrial membrane dynamics, mitochondrial protein unfolding reactions, and mitophagy [88].

A growing body of research has shown that, contrary to intuition, mitochondrial variants can sometimes extend lifespan. This has significantly changed the way we view mitochondria, as they are not just bioenergy factories, but also serve as signaling platforms for cellular homeostasis and organismal health maintenance [37]. Alternatively, the C5178A mutation in ETC complex I has been identified in centenarians, and the G9055A and the A10398G polymorphisms in the ATP6 and ND3 genes, respectively are associated with protective effects in PD [89]. ROS-induced damage to mtDNA is considered a major cause of aging [22,29]. The accumulation and overproduction of Aβ in mitochondria can accelerate mitochondrial dysfunction, such as mtDNA mutations, oxidative stress, and reduced mitochondrial axonal transport [82,83] contributing to neurodegenerative diseases such as AD [26,81,83]. Studies in animal models with AD suggest that Aβ-induced cytotoxicity reduces AHN prior to the emergence of a cognitive decline phenotype [35,56,81]. In patients, Aβ-related neuropathologies orchestrate neuroinflammation, neuronal cell death, mitochondrial dysfunction, and impaired AHN, that can lead to cognitive deficits and memory loss [56,83].

Ginsenosides have been shown to preserve mitochondrial functions [25,28,29,36]. For example, ginsenoside Rg3 mediated anti-aging effect to restore mitochondrial ATP and reduced the production of ROS via antioxidant proteins expression such as Nrf2 and HO-1 [36]. Supplementation with ginsenoside Rh2 increased antioxidant capacity of SOD1, and enhanced the expression of anti-aging and mitochondrial biogenesis-related genes peroxisome proliferator-activated receptor coactivator 1-α (PGC-1α) and SIRT1 [90]. PG berry extract (GBE) and soluble whey protein hydrolysate (WPH) improved mitochondrial biogenesis and increased mitochondrial numbers in muscle via PI3K/Akt pathway activation in aged mice [46]. Recently it has been revealed that ginsenoside significantly boost mitochondrial biogenesis and operational efficiency, influencing cellular energy processes. For instance, Ginseng Berry Concentrate treatment has been to shown to increase the expression of 561 genes coding for mitochondrial proteins, representing an upregulation of 50–60 % of nDNA-encoded mitochondrial genes. These upregulated genes are essential for mitochondrial biogenesis, oxidative phosphorylation (OXPHOS), and quality control mechanisms, including mitophagy [91]. Ginsenoside Rg3 prevented cognitive decline, memory impairment, and mitochondrial dysfunction in AD rats through anti-apoptotic pathways [92]. NFP improved cognitive function in aged brains and alleviated AD symptoms, suggesting its potential as a therapeutic candidate for age-related conditions [35]. Arginyl-fructosyl-glucose, a non-saponin ginsenoside, significantly attenuated mitochondrial dysfunction caused by D-Gal-induced ROS production, thereby delaying brain aging [25]. Consistently, SPJ improved mitochondrial morphology and reduced cell senescence via upregulation of mitofusin 2 and optic atrophy 1 expression levels and lowering the activity of dynamin-like protein 1 in the hippocampus of aging rats [21]. Ginsenoside Rg2 improved mitochondrial homeostasis in D-Gal-induced aging brains by promoting the degradation of the autophagy substrate p62 through the activation of LAMP2/TFEB expression [29]. Furthermore, Rg2 alleviated D-Gal-induced memory impairment and choline dysfunction by restoring redox balance and downregulating aging-related proteins such as p53, p21, and p16INK4α [28].

During aging, mitochondrial dysfunction can accelerate oxidative damage, which contributes significantly to cardiomyocyte apoptosis [93]. *Panax notoginseng* saponins (PNS) and SPJ in aging rats inhibited oxidative damage to attenuate cardiomyocyte apoptosis, and improved mitochondrial dysfunction in a dose-dependent manner. This effect is primarily achieved by upregulation of Forkhead Box Transcription Factor (FoxO3a) and Mn-SOD and inhibition of the PGC-1α, LC3β, and Beclin-1 levels [8,93]. Additionally, SPJ increased the expression levels of Ca2+-ATPase and Ca2+/Mg2+-ATPase, Na+/K + -ATPase, SOD, GSH-Px to confer neuroprotection and decreased MDA content in the cortex of aging rats. SPJ also increased protein expression of FoxO3a, SOD2, microtubule-associated protein light chain 3, Beclin1, and SIRT1 protein expression, while decreasing acetylated level of PGC-1α in the cortex and hippocampus of aging rats [23]. Remarkably, SPJ upregulated the expression of antioxidant genes, including Mn-SOD, HO-1, NAD(P)H quinone oxidoreductase 1 (NQO1), glutamate-cysteine ligase catalytic subunit (GCLC), Nrf2, and SIRT1 in the hippocampus, and reduced lipofuscin accumulation in the brain of D-Gal-induced aging rats [52]. Ginsenoside Rh4 enhanced mitochondrial homeostasis, delaying oxidative damage and skeletal muscle aging by modulating the PGC-1α-TFAM and HIF-1α-c-Myc pathways through activation of SIRT1 signaling [94]. In line with these findings, GBE + WPH mixture significantly improved muscle regeneration through PI3K/Akt pathway activation [46]. Chronic administration of *Panax quinquefolium* has been shown to prevent age-related lipid peroxidation and oxidative damage in the heart and muscles of rats [45]. Furthermore, Ginseng oligopeptides (GOPs) attenuated the oxidative stress-induced cell instability in HUVECs by increasing cell viability, enhanced telomerase activity, suppressing inflammatory responses, and protecting mitochondrial function thereby potentially contributing to a healthier lifespan [37].

Recently, mitochondrial ETC complex is proposed to play a critical role in the regulation of immune function. The ETC utilizes oxygen to generate ATP and regulates certain immune functions. For instance, activation of the NLRP3 inflammasome relies on an intact ETC to generate ATP. Complex II is essential for Th1 cell proliferation and, in certain cases, the production of effector cytokines, while complex III is vital for the proper function of Treg cells. In contrast, in B lymphocytes, OXPHOS and Complexes I, IV, and V are necessary to maintain lymphocyte proliferation and antibody production, with OXPHOS also playing a key role in regulatory B cell function [95].

Ginsenoside Rg1 pretreatment significantly improved mitochondrial function, including the activity of P16 and P21, as well as mitochondrial complex IV, and delayed premature senescence in human diploid fibroblast WI-38 cells [11]. Furthermore, ginsenoside Rb2 reversed cellular senescence by activating autophagy through the AMPK-mTOR pathway [96] suggesting that both Rg1 and Rb2 could be potential agents for rejuvenating human fibroblasts.

### 2.5 Aging, microbiota and gut-brain axis

In general, aging is regulated by oxidative stress responses and inflammatory processes, which in turn disrupt immune homeostasis and contribute to the gut microbiota disorders [30,34,54]. As a result, strategies to combat aging may focus on regulating the structure, function, and composition of the gut microbiota [9,30] which progressively changes with age-related diseases [30,34]. These disruptions in turn can promote the release of proinflammatory cytokines [30,34,97]. Thus, the key to improve immune function lies in maintaining a healthy gut microbiota composition, which in turn favorably affects host health and aging by enhancing antioxidant activity. Natural aging, an inevitable life process driven by systemic inflammation, results in the gradual decline of vital organs, including the gut and brain [1,2].

Emerging evidences suggest a close association of ginseng immunomodulatory effects on the gut microbiota regulation aiding in the repair of age-related damage and promoting health and longevity [9,34]. Ginseng improved intestinal stem cell (ISC) function in D-Gal-induced aging mice by activating the Wnt/β-catenin signaling pathway, delaying excessive proliferation of Lgr5 and Olfm4 cells [98]. Ginseng and its ginsenosides, due to their influence on gut microbiota regulation, are known to exhibit notable anti-aging effects [16,30,34,54]. Both PG and RG treatments alleviated the composition of pathogenic microbiota in D-Gal-induced aging mice to dominant beneficial groups such as Firmicutes, Bacteroidetes, Verrucomicrobia, and Proteobacteria, indicating that PG and RG have a stronger antagonistic effect [34]. Similarly, PG- and RG-treated groups increased beneficial bacteria, such as Bacteroidetes, Bifidobacteria, and *Lactobacillus* in aged individuals [34]. Probiotics like *Bifidobacterium* and *Lactobacillus* attenuarted oxidative stress induced by D-Gal [9,34]. Polysaccharides from ginsenoside residues (GRP) enhanced gut microbiota by increasing beneficial bacteria like Bacteroidaceae and Prevotellaceae, as well as the *Akkermansia* and *Bacteroides* genera, thus extending the lifespan of *C. elegans* [38]. *Akkermania*, a beneficial bacterium is known to possess notable anti-aging effect. Both RG and PG treatments increased the abundance of *Akkermansia* in D-Gal-induced aging, potentially preserving cognitive function, reducing oxidative stress, and maintaining gut microbiota composition and diversity [34]. FMT in ginseng-supplemented aged mice increased biodiversity and abundance of beneficial bacteria such as *Bacteroides, Dubosiella*, and Lachnospiraceae while suppressing potential pathogenic bacteria like *Helicobacter* and *Lachnoclostridium* [30]. However, future studies involving *in vivo* experiments administering these beneficial microbiomes directly to aging mice, as well as clinical trials with ginseng supplementation among elderly individuals, to further investigate the efficacy, mechanisms, and conclusive evidence of the anti-aging properties of ginseng.

PG prolonged the lifespan and enhanced the antioxidant capacity of *C. elegans* by increasing the relative abundance of beneficial bacteria and the activities of antioxidant enzymes such as CAT, GSH-Px, T-SOD, while reducing MDA and ROS levels [32]. Pretreatment with *Panax notoginseng* polysaccharides increased CAT and SOD activity and reduced MDA levels, indicating a prolonged survival rate and strong anti-aging effects in *C. elegans* [39]. Furthermore, ginseng volatile oil induced longevity and lifespan extension in *C. elegans* through antioxidant activity and autophagy activation, highlighting its potential as an anti-aging agent [99]. TGS improved the survival and longevity of *C. elegans* by enhancing lipid metabolism, reducing lipofuscin accumulation, and lowering ROS activity [40]. GRP enhanced antioxidant SOD capacity, reduced ROS activity, and diminished lipofuscin levels, thus extending the lifespan of *C. elegans* [38]. There is a growing concern to highlight the potential role of ginseng in regulating microbial diversity to modulate brain function and behavior via the microbiota-gut-brain axis in aged models [9,30]. GG and FG have been shown to enhance the relative abundance of beneficial Lactobacillus bacteria via the microbiota-gut-brain axis [9].

PG using FMT restored the gut microbial composition, to attenuate oxidative brain damage primarily via the regulation of microbiota-gut-brain axis in aged mice [30]. This study also indicated an increase in the relative abundance of beneficial microbiota such as *Bacteroides*, *Faecalibaculum*, Lachnospiraceae, and Muribaculaceae, while reducing the composition of pathogenic bacteria, including Bacteroidota and Proteobacteria [30]. Lachnospiraceae is recognized as an anti-aging beneficial microbiome of longevity [97]. Furthermore, the relative abundance of Lachnospiraceae is positively correlated with the production of short chain fatty acids (SCFAs) responsible to upregulate anti-inflammatory cytokines [3,30,97]. Ginseng content has been shown to increase the concentrations of SCFA, such as acetic acid, propionic acid and butyric acid in D-Gal-induced aging mice [98].

Taken together, this literature strongly supports the hypothesis that ginseng supplementation attenuated age-related pathologies via the activation of Treg cells expression, and the restoration of mitochondrial biogenesis by regulating antioxidant genes mechanism thus contributing to a healthy lifespan.

## 3 Conclusion

Aging is an unconscious inevitable biological process characterized by gradual, irreversible alterations that leads to the deterioration and decline of cellular, molecular, and organ functions, ultimately resulting in disease and death. Ginseng is recognized as a potent medicinal herb to promote health, extend lifespan, and strengthens the immune system of the host. Given a close correlation between aging populations and organ dysfunction, this review emphasizes the potential of ginseng as a therapeutic intervention for age-related diseases (Fig. 1, Fig. 2 and Table 1). Ginseng and its active components, ginsenosides, can improve organ function and overall quality of life by mitigating mitochondrial dysfunction, inflammatory cytokine effects, and oxidative stress through various mechanisms, including the maintenance of mitochondrial health, regulation of gut microbiota, modulation of age-related gene expression, and maintenance of immune cell populations. Despite these promising findings, the anti-aging effects of ginseng are still being explored. Therefore, additional preclinical and clinical research is necessary to develop safe, effective, and refined ginseng-based products for potential use in anti-aging therapies.

## Author contributions

HI and DKR collected, analyzed, and reviewed the literature, and wrote the main manuscript. HI and DKR prepared the Figures and Tables.

## Declaration of conflict of interest

The authors declare that they have no conflict of financial interests or personal relationships that could have appeared to influence the work reported in this manuscript.
